# Draft Genome Sequence of Strain JLT2015^T^, Belonging to the Family *Sphingomonadaceae* of the *Alphaproteobacteria*

**DOI:** 10.1128/genomeA.00226-13

**Published:** 2013-05-09

**Authors:** Kai Tang, Kesao Liu, Shuhui Li, Nianzhi Jiao

**Affiliations:** State Key Laboratory of Marine Environmental Science, Institute of Marine Microbes and Ecospheres, Xiamen University, Xiamen, China

## Abstract

Strain JLT2015^T^ was isolated from the southeastern Pacific, as a representative of a new genus of the family *Sphingomonadaceae* of the *Alphaproteobacteria*. Here, we present the draft genome sequence of strain JLT2015^T^, which provides insight into the oligotrophic strategy of this organism.

## GENOME ANNOUNCEMENT

Strain JLT2015^T^ (LMG27364, CGMCC1.12401) was isolated from surface waters (depth, 100 m) in the southeastern Pacific using previously described dilution-to-extinction culturing with rich organic medium (1). The phylogenetic analysis using 16S rRNA gene sequences showed that strain JLT2015^T^ was most closely related to a member of the family *Sphingomonadaceae*, *Novosphingobium pentaromativorans* (2), with 94.7% sequence similarity. Members of the *Sphingomonadaceae* are characterized by a smaller cell size than copiotrophic bacteria and display an oligotrophic strategy with low rates of growth in the nutrient-limited marine environment (3). Here, we report the draft genome sequence of strain JLT2015^T^.

The nucleotide sequence was determined using a 454 GS FLX sequencer. Assembly was performed using the Newbler 2.3 program. A total of 194,762 reads were obtained, which represented a 32.8-fold coverage of the genome. The strain JLT2015^T^ draft genome sequence contained 3,253,993 bp distributed in 38 contigs, with a G+C content of 64.2%. The assemblies were uploaded to the automated annotation platform on the Rapid Annotation using Subsystems Technology (RAST) server (4). A total of 3,058 protein-coding sequences with an average length of 950 bp were determined, occupying 89.33% of the genome. Among all the proteins predicted to be encoded, 2,370 proteins (77.5%) were assigned to different Clusters of Orthologous Groups (COG) categories (5). In addition, 1,494 genes (53.4%) were involved in 173 different pathways. The genome contained 48 tRNA genes and 4 rRNA operons.

Genes putatively encoding a carbon monoxide dehydrogenase were present in the genome. Genes encoding proteins involved in biosynthesis and export of extracellular or capsular polysaccharides were identified. Strain JLT2015^T^ contained abundant TonB-dependent transporter genes in the genome, which allow bacteria to take up scarce resources from nutrient-limiting environments (6). Strain JLT2015^T^ was predicted to have complete Embden-Meyerhof-Parnas, pentose-phosphate, and Entner-Doudoroff pathways. Strain JLT2015^T^ possessed genes responsible for assimilation of *N*-acetylglucosamine as a carbon source. Genes for a high-affinity phosphate transporter and a *pho* regulon for sensing of environmental inorganic phosphate availability were present. Genes for ammonium transporters, nitrate reductase, and sulfate reductase were also present in the strain JLT2015^T^ genome.

Comparison with genome sequences available in RAST (4) showed that *Sphingomonas* sp. strain SKA58 (score, 501), *Sphingomonas wittichii* RW1 (score, 490), and *Sphingobium japonicum* UT26S HTCC2559 (score, 462) were the closest neighbors of strain JLT2015^T^.

### Nucleotide sequence accession numbers.

The strain JLT2015^T^ shotgun genome sequence has been deposited at DDBJ/EMBL/GenBank under the accession number AMRV00000000. The version described in this paper is the first version, AMRV01000000.
